# Analysis of a single-institution cohort of patients with Felty's syndrome and T-cell large granular lymphocytic leukemia in the setting of rheumatoid arthritis

**DOI:** 10.1007/s00296-020-04757-4

**Published:** 2020-12-05

**Authors:** Vadim Romanovich Gorodetskiy, Yulia Vladimirovna Sidorova, Natalia Alexandrovna Kupryshina, Vladimir Ivanovich Vasilyev, Natalya Alexandrovna Probatova, Natalya Valerievna Ryzhikova, Andrey Borisovich Sudarikov

**Affiliations:** 1 Department of Intensive Methods of Therapy, V.A. Nasonova Research Institute of Rheumatology, Kashirskoye shosse 34A, Moscow, 115522 Russia; 2 Laboratory of Molecular Hematology, National Research Center for Hematology, Moscow, Russia; 3 Hematopoiesis Immunology Laboratory, N.N. Blokhin Russian Cancer Research Center, Moscow, Russia; 4 Diagnostic center of the MEDSI Clinic, Moscow, Russia; 5 Department of Pathology, N.N. Blokhin Russian Cancer Research Center, Moscow, Russia

**Keywords:** Felty’s syndrome, Large granular lymphocyte leukemia, Rheumatoid arthritis, STAT3, STAT5b, Comorbidity

## Abstract

T-cell large granular lymphocytic leukemia (T-LGLL) is a lymphoproliferative disorder characterized by a persistent increase in the number of large granular lymphocytes (LGLs), neutropenia, and splenomegaly. Clinical manifestations of T-LGLL in the setting of rheumatoid arthritis (RA) are often identical to those in which one would suspect Felty's syndrome (FS). These disorders are distinguished by the presence of T-cell clonality, which is present in T-LGLL but not in FS. Mutations in the signal transducer and activator of transcription 3 (*STAT3*) and 5b (*STAT5b*) genes can be used as molecular markers of T-LGLL, but their prevalence in FS is unknown.

Eighty-one patients with RA and unexplained neutropenia or/and an increase in the number of LGLs above 2 × 10^9^/L were stratified into RA-associated T-LGLL (*N* = 56) or FS (*N* = 25) groups based on the presence or absence of T-cell clonality. *STAT3* and *STAT5b* gene mutations were assessed in each group by means of allele-specific polymerase chain reaction assays. Clinical, immunological, laboratory data and the results of immunophenotyping of blood and bone marrow lymphocytes were also evaluated.

Mutations of the *STAT3* gene and an increase in the number of LGLs above 2 × 10^9^/L were detected in RA-associated T-LGLL, but not in FS (39% vs 0% and 21% vs 0%, respectively). Mutations in the *STAT5b* gene were not observed in either group. Expression of CD57, CD16, and CD5^−/dim^ on CD3^+^CD8^+^ T-lymphocytes was observed in both RA-associated T-LGLL and FS.

*STAT3* gene mutations or LGL counts over 2 × 10^9^/L in RA patients are indicative of T-LGLL.

**Supplementary Information:**

The online version contains supplementary material available at 10.1007/s00296-020-04757-4.

## Introduction

Felty’s syndrome (FS) is a clinical diagnosis that is suspected in patients with rheumatoid arthritis (RA), neutropenia, and splenomegaly. The persistence of unexplained neutrophil counts of less than 1.5–2.0 × 10^9^/L is a mandatory criterion for diagnosing FS. Although splenomegaly is a component of the triad for defining FS, currently, it is not an absolute requirement for diagnosis [1, 2]. The vast majority of patients with FS have high titers of both rheumatoid factor (RF) and anti-cyclic citrullinated peptide (anti-CCP) antibodies [3, 4]. FS usually develops, on average, 12 years after RA presentation; the mean age of the patients is 60 years, with a female-to-male ratio of 1.5:1 [5]. There is no specific diagnostic test to confirm the presence of FS; therefore, the identification of FS is essentially based on an exception diagnosis.

T-cell large granular lymphocytic leukemia (T-LGLL) is a rare chronic lymphoproliferative disorder characterized by the expansion of clonal, immunophenotypically distinct, large granular lymphocytes (LGLs). In most cases, T-LGLL is characterized by an immunophenotype of cytotoxic T-lymphocytes (CD3^+^/CD8^+^) that co-express the natural killer (NK)-cell lineage-associated antigens CD16 and/or CD57, and diminished or absent expression of the pan-T-cell markers CD5 and/or CD7 [6, 7]. Approximately 15% of patients with T-LGLL have RA [8, 9]; on the contrary, the clonal expansion of LGLs was detected in 3.6% of patients with RA [10]. Human leukocyte antigen (HLA)-RD4 is detected in approximately 90% of patients with T-LGLL and concurrent RA, but in only 33% of patients with T-LGLL without RA (similar to that in the general population); this strongly supports an immunogenetic link between T-LGLL and RA [11]. T-LGLL is usually diagnosed 10–20 years after the manifestation of RA [5], although both conditions may present simultaneously, and T-LGLL may also precede the clinical manifestations of RA by several years [12–15]. A typical manifestation of T-LGLL includes neutropenia and splenomegaly, which are detected in up to 84% and 50% of patients, respectively [16]. Historically, a definitive diagnosis of LGLL can be made if the LGL count is persistently greater than 2 × 10^9^/L in peripheral blood for more than 6 months [16, 17]. However, T-LGLL can currently be diagnosed if the LGL count exceeds 0.4 or 0.5 × 10^9^/L, provided that a clonal T-cell population is found with an appropriate clinical context (cytopenia and/or an autoimmune disease) [18–20]. Bone marrow involvement is present in at least 75% of T-LGLL cases, though it is often subtle and difficult to detect morphologically. Although specific criteria have been proposed for the diagnosis of T-LGLL in bone marrow sections using immunohistochemistry [21], they do not seem to be sufficiently specific to distinguish T-LGLL from FS [5].

Currently, T-LGLL and FS can be distinguished by T-cell clonality, which can be determined by assessing the T-cell receptor (TCR) gene rearrangements present in T-LGLL, but not in FS [5, 13, 22]. In addition, recent studies have identified activating somatic mutations in the signal transducer and activator of transcription 3 (STAT3) gene in 27–72% of patients with T-LGLL [23–26] and in the signal transducer and activator of transcription 5b (STAT5b) gene in 2% of patients with T-LGLL [27]. However, the prevalence of *STAT3* and *STAT5b* mutations in FS is unknown.

In this study, we stratified 81 patients with RA and unexplained neutropenia or/and an increase in the number of LGLs into two groups (RA-associated T-LGLL or FS) based on the presence or absence of T-cell clonality; we then examined *STAT3* and *STAT5b* gene mutations in both groups. We also assessed the clinical, immunological, and laboratory data of the patients, as well as the results of immunophenotyping of blood and bone marrow lymphocytes of those with FS and RA-associated T-LGLL to identify characteristics that would allow for their differential diagnosis.

## Patients and methods

This retrospective study included 81 adult patients who met the following two criteria: (i) RA diagnosed according to the 2010 American College of Rheumatology/European League Against Rheumatism criteria [28]; (ii) neutropenia (absolute neutrophil counts less than 1.5 × 10^9^/L) or/and an elevated LGL count exceeding 2 × 10^9^/L in peripheral blood. Patients with drug-induced neutropenia were excluded from the study.

Tissue samples available for the studies are shown in Table 1. Peripheral blood smears were re-examined for LGL counting in 64 cases. Bone marrow aspiration with differentiated cell counts was performed in 53 cases; a bone marrow biopsy was also performed in 45 of these. The collected clinical data included patient age, sex, presence of splenomegaly, RA duration, titers of RF, anti-CCP antibodies, antibodies against mutated citrullinated vimentin (anti-MCV), antinuclear antibodies (ANA), erosive arthritis, and associated Sjögren syndrome (SS).

**Table 1:** Tissue samples available for the studies

		N (FS)	N (RA with T-LGLL)	Ʃ
PB smears for LGL counting		17	47	64
BM aspirates for differentiated cell count		13	40	53
BM histological examination		11	34	45
BM immunohistochemical study		10	28	38
T-cell clonality	PB	14	30	44
	BM	1	0	1
	PB + BM	8	15	23
	PB + Spleen	1	5	6
	BM + Spleen	0	2	2
	PB + BM + Spleen	1	4	5
*STAT3* and *STAT5b* gene mutations	PB	19	39	58
	BM	1	1	2
	PB + BM	2	5	7
	PB + Spleen	2	7	9
	BM + Spleen	0	1	1
	Spleen	0	3	3
	ND	1	0	1
Flow cytometric analysis	PB	15	36	51
	BM	1	3	4
	PB + BM	2	15	17

*N* available number of samples, *FS* Felty's syndrome, *T-LGLL* T-cell large granular lymphocytic leukemia, *PB* peripheral blood, *BM* bone marrow, *LGL* large granular lymphocytes, *RA* rheumatoid arthritis, *ND* no data

### Flow cytometric analysis

A four-color flow cytometric analysis was performed on specimens of peripheral blood alone (51 cases), peripheral blood and bone marrow (17 cases), or bone marrow alone (four cases). Lymphocytes were gated using CD45 versus side-scatter dot-plots. Cells were stained with a panel of fluorescence-labeled monoclonal antibodies, including CD3, CD4, CD5, CD7, CD8, CD16, CD19, CD57, TCR-α/β, and TCR-γ/δ. Flow cytometric analysis was performed on a BD FACSCanto™ II (Becton Dickinson, San Jose, CA, USA) system using FCS Express version 3 (De Novo Software, Los Angeles, CA) software.

### Immunohistochemical studies

Immunohistochemical studies were carried out in 38 of 45 cases using sections of decalcified paraffin-embedded bone marrow biopsy specimens. The following antibodies were used at the dilutions suggested by the manufacturers: CD3 (polyclonal, Dako), CD4 (clone 4B12, Dako), CD8 (clone C8/144B, Dako), CD20 (clone L26, Dako), CD56 (clone 123C3, Dako), CD57 (clone TB01, Dako), granzyme B (clone GrB-7, Dako), and T-cell restricted intracellular antigen 1 (TIA-1) (clone 2G9, Immunotech, France). After dewaxing and heat-induced antigen retrieval, immunostaining was performed on an Autostainer Link 48 (Dako, Denmark) according to the manufacturer’s instructions. All immunostained samples were counterstained with hematoxylin.

### Evaluation of STAT3 and STAT5b gene mutations, and T-cell clonality

*STAT3* and *STAT5b* gene mutations, and T-cell clonality were examined using genomic DNA extracted from blood, bone marrow, and spleen tissue samples (Table 1). The evaluation of T-cell clonality based on the rearrangements of the TCR gamma (Vγ–Jγ) and TCR beta (Vβ–Jβ, Dβ–Jβ) genes was performed in all cases. In addition, TCR delta (Vδ-Dδ-Jδ) gene rearrangement was assessed in all cases with suspected γδ T-cell proliferation. T-cell clonality assays were performed according to the BIOMED-2 standardized protocol [29]. Polymerase chain reaction (PCR) amplification was carried out using an automated DNA Engine thermocycler (BioRad, Hercules, USA), and fragments were detected using an ABI PRISM 3130 Genetic Analyzer (Applied Biosystems, Foster City, CA); the data were analyzed using GeneMapper software version 4.0 (Applied Biosystems, Foster City, CA).

Allele-specific (AS) TaqMan Real-Time PCR assays were developed to determine the somatic point mutations in the *STAT3* (p.Y640F; p.N647I; p.D661V; p.D661Y; p.D661H; p.D661N) and *STAT5b* (p.N642H) genes. DNA (200–400 ng) was added to 25 μL of the reaction mixture containing 10 pmol of WT (wild type)-specific or MT (mutated type)-specific forward primer, 10 pmol of common reverse primer, and 7.5 pmol of the fluorescent probe. AS-PCR was then performed in triplicate (three WT & three MT) using a StepOne Real-Time PCR System (Applied Biosystems, USA). PCR conditions included preliminary denaturation at 95 °C for 5 min, followed by 45 cycles at 95 °C for 30 s, 62 °C for 30 s, and 72 °C for 30 s. A mixture of DNA from healthy donors was used as a negative control. Samples with mutations confirmed by Sanger sequencing were used as positive controls. The primer and probe sequences are shown in Supplement 1. Patients with T-cell clonality were assigned to the T-LGLL group; in the absence of T-cell clonality, they were assigned to the FS group.

### Statistical analysis

Statistical analyses were performed using R software (version 3.5). Descriptive statistics are presented as numbers and percentages for categorical data, and as medians and ranges for continuous data. Differences in the distributions of categorical variables among patients with FS and RA with T-LGLL were assessed by two-sided proportion test with continuity correction and 95% confidence interval; continuous variables were assessed by the Mann–Whitney *U* test. *P* values ≤ 0.05 were considered statistically significant.

## Results

Of the 81 RA patients included in this study, neutropenia of less than 1.5 × 10^9^/L was detected in 78 cases. In seven of these 78 cases, there was also an increase in the absolute number of circulating LGLs exceeding 2 × 10^9^/L. According to the absence or presence of T-cell clonality, these 78 patients were stratified into two groups, either the FS (25 patients) or the RA-associated T-LGLL (53 patients) group, respectively. In three out of 81 patients, neutropenia was absent. However, there was an increase in the number of T-LGLs in blood exceeding 2 × 10^9^/L and T-cell clonality, which allowed classifying as T-LGLL. Thus, this study included 25 patients with FS and 56 patients with RA-associated T-LGLL.

### FS cohort

**Fig. 1:**
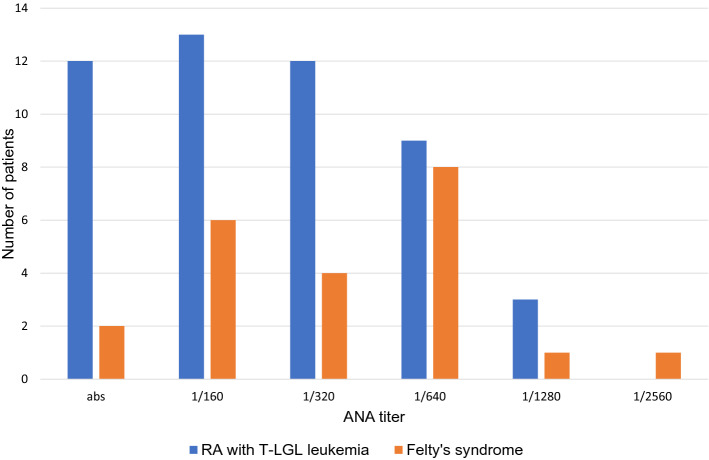
The results of the ANA test in the patients with FS and RA with T-LGLL

The clinical and biological characteristics of the 25 patients with FS are presented in Table 2 and Supplement 2. Nineteen (76%) patients were female and eight (32%) had concomitant SS. The median age at FS diagnosis was 56 years (range 30–79 years). The median duration of RA prior to FS diagnosis was 7 years (range 0–35 years). Erosive arthritis at the time of FS diagnosis was detected in 17 (77%) of 22 patients. RF, anti-CCP and anti-MCV antibodies were positive in 23 (92%) of 25, in 23 (96%) of 24, and in 12 (100%) of 12 patients, respectively. In two RF-negative patients, the anti-CCP and anti-MCV titers were highly positive. The ANA test was positive in 20 (91%) of 22 patients (Fig. 1).

**Table 2:** Characteristics of patients with Felty's syndrome and RA with T-LGL leukemia

Clinical and biological features	Felty's syndrome	RA with T-LGLL	*P* ≤ 0.05	95% CI	
				Min	Max
Number of patients	25	56			
No. female/no. male	19/6	41/15			
*Age (y), median (range)	56 (30–79)	58.5 (27–76)			
^#^Duration (y) of RA, median (range)	7 (0–35)	6 (0–36)			
Erosive arthritis	77% (17/22)	69% (35/51)			
RF positive	92% (23/25)	82% (46/56)			
Anti-CCP positive	96% (23/24)	88% (49/56)			
Anti-MCV positive	100% (12/12)	81% (34/42)			
Splenomegaly	83% (19/23)	56% (31/55)	0.05	0.029	0.496
Associated Sjögren’s syndrome	32% (8/25)	20% (11/56)			
*STAT3* mutation positive	0% (0/24)	39% (22/56)	0.0009	− 0.551	− 0.235
*STAT5b* mutation positive	0% (0/24)	0% (0/56)			
Hematologic features					
Leukocytes (× 10^9^/L), median (range)	1.900 (1.100–3.400)	2.650 (0.700–10.200)	0.023		
Neutrophils (× 10^9^/L), median (range)	0.496 (0.052–1.224)	0.495 (0.000–3.468)			
Neutrophils (× 10^9^/L) < 0.5	52% (13/25)	52% (29/56)			
Lymphocyte (× 10^9^/L), median (range)	1.092 (0.420–2.320)	1.752 (0.490–7.452)	0.0005		
Lymphocyte (× 10^9^/L) > 4.0	0% (0/25)	14% (8/56)	0.05	− 0.263	− 0.022
LGLs (× 10^9^/L), median (range)	0.418 (0.117–1.036)	0.850 (0.252–6.552)	0.0004		
LGLs (× 10^9^/L) > 0.4	59% (10/17)	85% (40/47)			
LGLs (× 10^9^/L) > 2.0	0% (0/17)	21% (10/47)	0.05	− 0.370	− 0.056
Percent of lymphocytes in the BM	12.4 (3.8–18.4)	22.0 (5.2–80.3)	0.0025		
Immunophenotypic characteristics of cytotoxic (CD3 + CD8 +) T-lymphocytes					
CD57^+^	59% (10/17)	92% (49/53)	0.003	− 0.620	− 0.053
CD5^−/dim^	71% (12/17)	93% (50/54)	0.05	− 0.486	0.046
CD16^+^	7% (1/15)	24% (12/51)			

^*^at FS or T-LGLL diagnosis; # before the diagnosis of FS or T-LGLL, *RA* rheumatoid arthritis, *FS* Felty's syndrome, *T-LGLL* T-cell large granular lymphocytic leukemia, *BM* bone marrow, *LGLs* large granular lymphocytes

The median neutrophil count was 0.496 × 10^9^/L, with a range of 0.052–1224.0 × 10^9^/L. Absolute neutrophil counts less than 0.5 × 10^9^/L were observed in 13 (52%) of 25 patients. The absolute number of lymphocytes ranged from 0.42 to 2.32 × 10^9^/L, with a median of 1.092 × 10^9^/L. LGL counts in the blood exceeding 0.4 × 10^9^/L were observed in 10 (59%) of 17 cases, but the counts did not exceed 2.0 × 10^9^/L in any of the cases.

The results of the flow cytometric immunophenotyping of cytotoxic (CD3^+^CD8^+^) T-lymphocytes are summarized in Table 2. Absent or diminished expression of CD5 (CD5^−/dim^) was present in 12 (71%) of 17 cases. CD57 expression was found in 10 (59%) of 17 cases, while CD16 expression was found in only one (7%) of 15 cases.

**Fig. 2:**
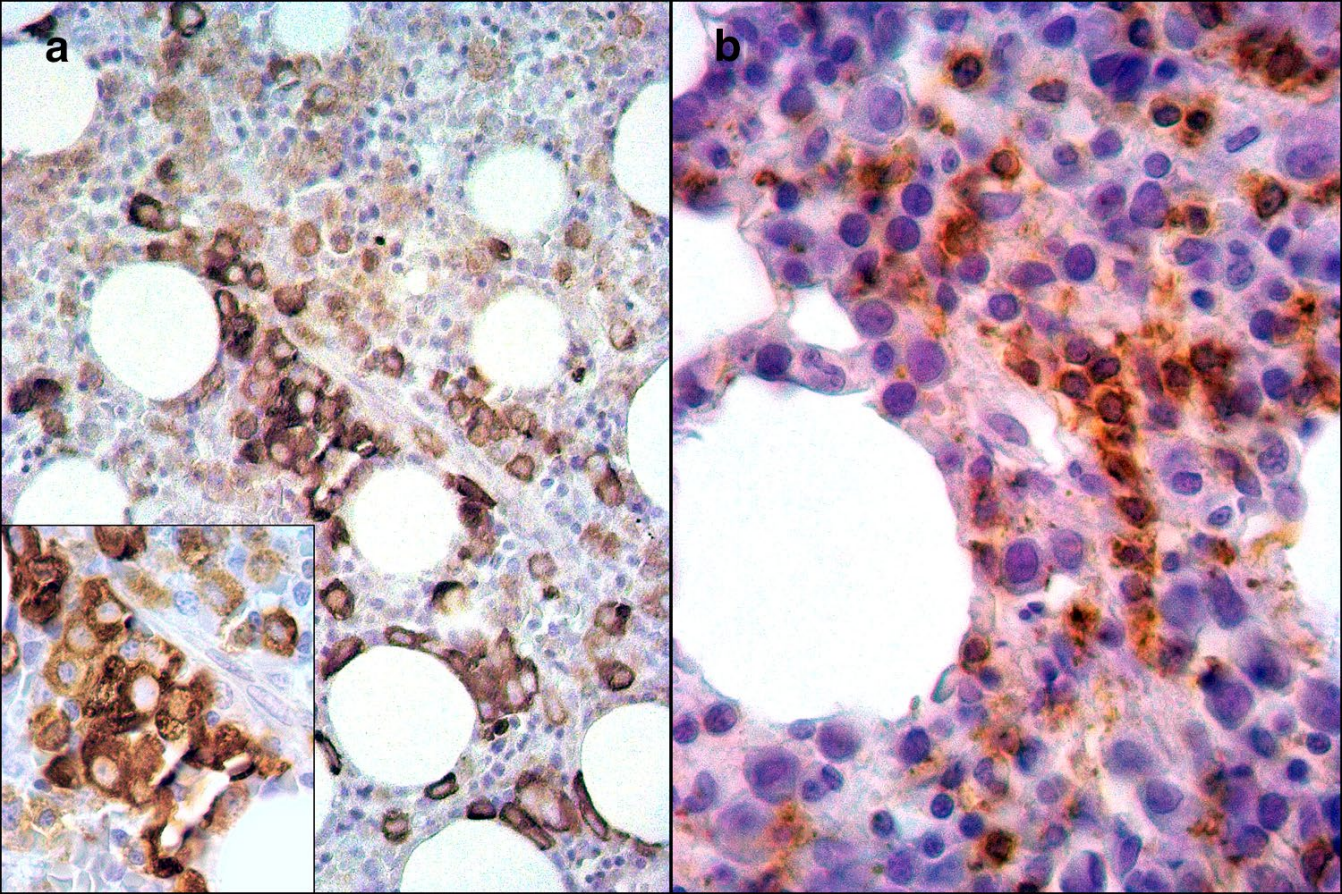
Examples of bone marrow findings in the patients with FS. **a** Patient #9. Immunoperoxidase staining for granzyme B highlights numerous granzyme B^+^ lymphocytes; original magnification: × 400. A cluster of more than 10 cells is shown in the inset with an original magnification of × 1000. **b** Patient #24. Immunoperoxidase staining for CD8 demonstrates a linear array of intravascular CD8^+^ lymphocytes; original magnification: × 1000

Bone marrow aspirate samples were available in 13 patients with FS. In all cases, there were no signs of myelodysplasia. The number of lymphocytes in the bone marrow did not exceed the upper limit of the adult norm (23.8% lymphocytes) in any case, and they comprised between 3.8 and 18.4% of all nucleated cells. Bone marrow aspirate differential cell counts showed a significant reduction in the number of segmented neutrophils in 10 of 13 patients; in one other patient, there was a reduction in the band and segmented neutrophils. However, in two patients, the bone marrow aspirate differential count was normal, despite a decrease in the number of neutrophils in the blood. Bone marrow immunohistochemistry showed interstitial clusters and/or linear arrays of intravascular CD8^+^/granzyme B^+^ lymphocytes in two of 10 cases (Fig. 2). Splenomegaly was observed in 19 (83%) of 23 patients. Mutations in the *STAT3* and *STAT5b* genes were not detected in any of the 24 patients with FS.

### RA with T-LGLL cohort

The clinical and biological characteristics of the 56 patients with RA-associated T-LGLL are presented in Table 2 and Supplement 3.

In a cohort of patients with RA-associated T-LGLL, 41 (73%) patients were female, and the median age at the time of T-LGLL diagnosis was 58.5 years (range 27–36 years). SS associated with RA was diagnosed in 11 (20%) patients. The median duration of RA prior to a T-LGLL diagnosis was 6 years (range 0–36 years). Erosive arthritis at the time of T-LGLL diagnosis was detected in 35 (69%) of 51 patients. RF was positive in 46 (82%) of 56 patients and was negative in 10 (18%) cases. Anti-CCP and anti-MCV were present in 49 (88%) of 56 patients and in 34 (81%) of 42 patients, respectively. Four patients had erosive seronegative RA. The ANA test was positive in 37 (76%) of 49 patients (Fig. 1).

The absolute number of neutrophils in peripheral blood ranged from 0 to 3.468 × 10^9^/L, with a median of 0.495 × 10^9^/L; in 29 (52%) of 56 patients, the absolute neutrophil count was less than 0.5 × 10^9^/L. The absolute number of lymphocytes in peripheral blood ranged from 0.490 to 7.452 × 10^9^/L, with a median of 1.752 × 10^9^/L; in eight (14%) of 56 patients, the absolute lymphocyte count exceeded 4 × 10^9^/L. An absolute number of LGLs exceeding 0.4 × 10^9^/L was observed in 40 (85%) of 47 cases; it exceeded 2.0 × 10^9^/L in 10 of these cases.

The results of the flow cytometric immunophenotyping of lymphocytes of patients with RA-associated T-LGLL are shown in Table 2. The most commonly identified phenotypic abnormalities on cytotoxic (CD3^+^CD8^+^) T-lymphocytes were CD57^+^ [in 49 (92%) of 53 cases] and CD5^−/dim^ [in 50 (93%) of 54 cases]. The expression of CD16 was found in 12 (24%) of 51 cases.

Bone marrow aspirate samples were available from 40 patients with T-LGLL. Dysplastic changes in myeloid cell lineages and megakaryocytes, as well as a marked decrease of erythroid cell lineages, were observed in case #41. Multilineage dysplasia was found in case #49. The number of lymphocytes in the bone marrow ranged from 5.2 to 80.3% of nucleated cells and exceeded the upper limit of the adult normal level in 15 (60%) of 25 cases. Bone marrow aspirate differential counts showed a significant reduction in segmented neutrophils in 15 patients, segmented and band neutrophils in three patients, segmented neutrophils, band neutrophils, and neutrophilic metamyelocytes in 11 patients, and segmented neutrophils, band neutrophils, neutrophilic metamyelocytes, and neutrophilic myelocytes in five patients. In six patients, the bone marrow aspirate differential count was normal, despite the presence of neutropenia in five of these patients. In 21 (75%) of 28 cases, immunohistochemical studies of the bone marrow showed interstitially distributed clusters of at least eight CD8^+^ or TIA-1^+^ lymphocytes or clusters of at least six granzyme B^+^ lymphocytes and/or linear arrays of intravascular CD8^+^, TIA-1^+^, or granzyme B^+^ lymphocytes corresponding to the involvement of T-LGLL. Splenomegaly was observed in 31 (56%) of 55 patients. *STAT3* gene mutations were found in 22 (39%) of 56 patients with RA-associated T-LGLL. The p.Y640F mutation was present in nine (41%) cases, followed by p.N647I and p.D661Y in six (27%) cases each, and p.D661V in one (5%) case (Supplement 3). No cases with more than one mutation were identified. A somatic p.N642H mutation in *STAT5b* gene was not found in any of the 56 patients with RA-associated T-LGLL.

## Discussion

The most typical presenting features of T-LGLL include neutropenia, an increase in the number of LGLs in the blood, and splenomegaly. In the setting of RA, cases of T-LGLL with low number of LGLs in the peripheral blood and with neutropenia are indistinguishable from FS; therefore, they represent a diagnostic challenge. At present, discriminating between FS and T-LGLL in the setting of RA often depends on the result of an assessment of T-cell clonality, which has well-known gray areas in the interpretation [30]. Moreover, there has been considerable discussion regarding the significance of dominant T-cell clones as a hallmark of T-cell malignancy because small populations of clonally expanded T-LGLs are revealed in healthy individuals and in situations associated with an exuberantly reactive response [31–35].

Mutations in the *STAT3* and *STAT5b* genes can be used as molecular markers for T-LGLL diagnostics [36], but their prevalence in FS and their diagnostic value for the differential diagnosis between FS and RA-associated T-LGLL remain unclear. We did not find *STAT3* mutations in any of the 24 patients with FS, as opposed to 22 of the 56 patients with RA-associated T-LGLL (0% versus 39%, *P* = 0.00086). Savola et al. examined *STAT3* and *STAT5b* mutations in 14 patients with RA and neutropenia [37]. Similar to our patient cohort, they did not find any *STAT5b* mutations. However, in contrast to our results, they did identify *STAT3* mutations in six (43%) of 14 patients. We believe that the difference between the outcomes reported by Savola et al. and our study can be attributed to the different methods of assessing T-cell clonality and the patient selection criteria. We tested T-cell clonality based on the rearrangement of gamma, beta, and, in cases with the γδ T-cell phenotype, also delta chain encoding genes by a PCR-based assay, whereas Savola et al. studied the clonality of T-cells by flow cytometry using a Vβ kit that covered only 70% of the Vβ T-cell repertoire. In addition, in contrast to Savola et al., we did not include patients with T-cell clonality in the FS group.

Differences in sex, the age at diagnosis, the duration of RA, the incidence of erosive arthritis, RF, anti-CCP, and anti-MCV positivity, and SS diagnosis between the groups with FS and RA-associated T-LGLL were statistically insignificant in our sample. Splenomegaly ranged from massive to detectable based solely on abdominal ultrasound and was more common in patients with FS than in those with RA-associated T-LGLL (83% versus 56%, *P* = 0.05).

**Fig. 3:**
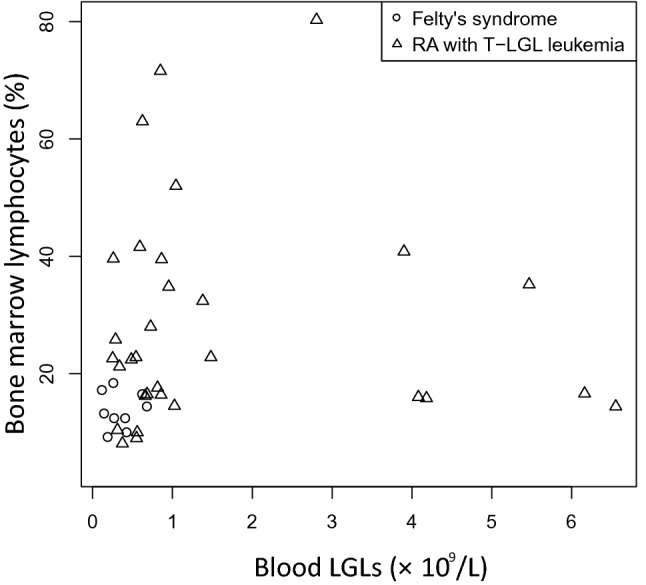
The distribution of FS and RA-associated T-LGLL cases according to the number of LGLs in the peripheral blood in relation to the percentage of lymphocytes in the bone marrow

In our patients with FS, the increase of absolute number of LGLs in the peripheral blood over 0.4 × 10^9^/L was detected in 10 (59%) of 17 cases, but it did not exceed 2.0 × 10^9^/L in any case. In the group of patients with RA-associated T-LGLL, 40 (85%) of 47 patients had more than 0.4 × 10^9^/L circulating LGLs in the blood and, in contrast to patients with FS, 10 (21%) of these 47 patients had an increase of LGLs in peripheral blood of more than 2.0 × 10^9^/L. A statistically significant difference was found between the patients with FS and RA-associated T-LGLL in terms of the percentage of lymphocytes in the bone marrow. The distribution of FS and RA-associated T-LGLL cases according to the number of LGLs in the peripheral blood in relation to the percentage of lymphocytes in the bone marrow is presented in Fig. 3. The bone marrow aspirate differential counts showed increased number of lymphocytes in 60% of patients with RA-associated T-LGLL, but no one in the FS group.

Flow cytometric immunophenotyping plays an important role in the diagnosis of T-LGLL; however, the value of this method in the differential diagnosis of T-LGLL and the reactive expansion of LGLs in FS has not been established. Statistically significant differences were found in the expression of the CD57 antigen and the aberrant (diminished or absent) expression of CD5 between the FS and RA-associated T-LGLL groups. In contrast, no statistically significant difference was found in the expression of CD16.

Morice et al. suggested that clusters of six or more granzyme B^+^ lymphocytes or sinusoidal linear arrays of CD8^+^, TIA-1^+^, or granzyme B^+^ lymphocytes are specific for T-LGLL, but not for non-clonal T-LGL disorders [21]. However, in our cohort, as in the Burks et al. study [5], there were patients with a specific pattern of bone marrow infiltration by cytotoxic T-lymphocytes that is characteristic of T-LGLL, but in the absence of T-cell clonality (Fig. 2). Further data accumulation is necessary to facilitate the interpretation of these results.

The clinical features of RA in the setting of T-LGLL have not been thoroughly characterized. According to Prochorec-Sobieszek et al., the severity of arthritis can vary [38]. It should be noted that several seropositive patients without a history of inflammatory arthritis had erosions and typical radiographic changes for RA.

The duration from the diagnosis of RA until the development of FS or T-LGLL in our cohort was shorter than that reported by others [5]. All 25 patients with FS were seropositive (RF^+^/anti-CCP^+^/anti-MCV^+^ or RF^−^/anti-CCP^+^/anti-MCV^+^), whereas in the RA group with T-LGLL, four of 56 patients had erosive, seronegative RA.

The percentage of patients in our cohort with T-LGLL with an increase in blood lymphocyte counts exceeding 4.0 × 10^9^/L was 14%, which is comparable to a study by Zhu et al. [39]. However, in earlier works, the authors reported a much higher percentage of such patients at 74% [16] and 51% [8]. This is probably because of changes in the criteria for T-LGLL diagnosis over time.

## Limitations

Since our study was retrospective, some data were not available. In addition, we used the AS TaqMan real-time PCR assay to detect somatic point mutations in the *STAT3* and *STAT5b* genes using a set of primers for the most common mutations in the two genes. Even though this approach provides much higher sensitivity than Sanger sequencing, some rare mutations that were not covered by the developed primers could not be identified.

It should be noted that in six cases (all with massive splenomegaly), a study of the spleen detected T-cell clonality that was absent in the blood and/or bone marrow. In addition, out of the 28 patients for whom both blood and bone marrow samples were tested, two (7%) displayed T-cell clonality only in the bone marrow and not in the blood. Given the above, it cannot be ruled out that among the 14 patients who were classified as having FS based on the absence of T-cell clonality only in the peripheral blood, there were still patients with T-cell clonality in the spleen and/or bone marrow.

## Conclusion

We did not detect *STAT3* gene mutations in any of the 24 patients with FS, whereas mutations were found in 22 (39%) of 56 patients with RA-associated T-LGLL. An increase in the absolute number of LGLs in the blood above 2 × 10^9^/L was also a specific characteristic of RA-associated T-LGLL that was not found in the FS group in our study. In contrast, typical immunophenotypic markers of T-LGLL were also found in the polyclonal expansion of cytotoxic T-lymphocytes in the setting of RA. Although further data are required, our results suggest that the currently used criteria for immunohistochemical examination of the bone marrow do not seem to be able to definitively distinguish FS from T-LGLL in the setting of RA.

## Supplementary Information

Below is the link to the electronic supplementary material.

Supplementary file1 (DOC 32 KB)

Supplementary file2 (DOC 72 KB)

Supplementary file3 (DOC 132 KB)

## Data Availability

All data generated or analyzed during this study are included in this article.
