# Supplementary material for: Analysis of a single-institution cohort of patients with Felty's syndrome and T-cell large granular lymphocytic leukemia in the setting of rheumatoid arthritis
Source: Rheumatol Int. 2020 Dec 5;41(1):147–56. doi: 10.1007/s00296-020-04757-4 (PMC7806571; doi:10.1007/s00296-020-04757-4)
Supplement: Supplementary file 1 — Supplementary file1 (DOC 32 KB) [file 296_2020_4757_MOESM1_ESM.doc]

**Supplement 1.** The sequences of allele specific primers and probes (5’-3’)

| Mutation | Forward primers | Reverse (common) primers and fluorescent probes |
| --- | --- | --- |
| STAT3 Y640F | WT: ATCCAGTCCGTGGAACCTT(A)  MT: ATCCAGTCCGTGGAACCTT(T) | CCAGTGGAAAGACACCAGGATATTG  FAM -AGCAGCAGCTGAACAACATGTCATTTGCTGA -BHQ1 |
| STAT3 N647I | WT: ATACACAAAGCAGCAGCTGAACtA  MT: ATACACAAAGCAGCAGCTGAACtT | TCCTCCTTGGGAATGTCAGGATAGA  FAM-AGACCAGTGGAAAGACACCAGGATATTGGTA-BHQ1 |
| STAT3 D661V | WT: AAATCATCATGGGCTATAAGATCATGG  MT: AAATCATCATGGGCTATAAGATCATtGT |
| STAT3 D661Y | WT: AAATCATCATGGGCTATAAGATCATGG  MT: AAATCATCATGGGCTATAAGATCAaGT |
| STAT3 D661H | WT: AAATCATCATGGGCTATAAGATCATGG  MT: AAATCATCATGGGCTATAAGATCAaGC |
| STAT3 D661N | WT: AAATCATCATGGGCTATAAGATCATGG  MT: AAATCATCATGGGCTATAAGATCAaGA |
| STAT5b N642H | WT: GTGGTAAAAGGCATCAGAT(T)  MT: GTGGTAAAAGGCATCAGAT(G) | TCCTAATTCAGAAATCATGTTTAG  FAM-GTGATTGTTCTGTTTATTGATCTAGAGG-BHQ1 |

Mismatched nucleotides are lowercased. Locked nucleic acid nucleotides are given in parentheses. WT, wild type; MT, mutated type
