# Supplementary material for: Analysis of a single-institution cohort of patients with Felty's syndrome and T-cell large granular lymphocytic leukemia in the setting of rheumatoid arthritis
Source: Rheumatol Int. 2020 Dec 5;41(1):147–56. doi: 10.1007/s00296-020-04757-4 (PMC7806571; doi:10.1007/s00296-020-04757-4)
Supplement: Supplementary file 2 — Supplementary file2 (DOC 72 KB) [file 296_2020_4757_MOESM2_ESM.doc]

**Supplement 2.** Characteristics of 25 patients with Felty's syndrome included in the study

| Patient no. / Sex / *Age (y) | *Duration  of RA (y) | Erosive arthritis | Splenomegaly | RF / anti-CCP / anti-MCV | Absolute  leukocyte count (×10^9^/L) | Absolute neutrophil count (×10^9^/L) | Absolute lymphocyte count (×10^9^/L) | Absolute LGL count (×10^9^/L) | Percent of lymphocytes in the BM | BM aspirate differential counting | Samples for T-cell clonality testing | Samples for STAT3 and STAT5b gene mutations testing |
| --- | --- | --- | --- | --- | --- | --- | --- | --- | --- | --- | --- | --- |
| 1. / M / 51 | 23 | + | + | + / + / + | 3.100 | 0.961 | 1.364 | 0.775 | ND | ND | PB | PB |
| 2. / M / 61 | 24 | + | ND | + / ND / ND | 2.700 | 1.053 | 1.323 | 0.810 | ND | ND | PB | PB |
| 3. / M / 79 | 0 | **−** | + | + / + / ND | 2.800 | 1.148 | 1.092 | 1.036 | ND | ND | PB | PB |
| 4. / F / 50 | 10 | + | + | + / + / ND | 2.200 | 1.100 | 0.880 | ND | 13.4 | ↓ SN | BM | BM |
| 5. / F / 59 | 6 | + | + | + / + / ND | 1.500 | 0.780 | 0.570 | 0.330 | ND | ND | PB | PB |
| 6. / F / 69 | 7 | + | + | + / + / + | 1.300 | 0.546 | 0.533 | 0.117 | 17.2 | ↓ SN | PB | PB |
| 7. / M / 67 | 35 | + | + | +/ + / + | 1.700 | 0.612 | 0.714 | 0.408 | 12.4 | ↓ SN | PB, BM | PB |
| 8. / F / 63 | 28 | + | − | + / + / + | 2.100 | 0.399 | 1.386 | 0.189 | 9.2 | ↓ SN | PB, BM | PB |
| 9. / F / 56 | 26 | **−** | + | + / + / ND | 1.300 | 0.052 | 1.079 | 0.260 | 18.4 | ↓ SN | PB, Spleen | PB, Spleen |
| 10. / F / 52 | 7 | ND | + | + / + / ND | 1.800 | 0.324 | 1.242 | 0.360 | ND | ND | PB | PB |
| 11. / F / 52 | 32 | + | + | + / + / ND | 1.600 | 0.192 | 0.848 | 0.656 | ND | ND | PB | PB |
| 12. / M / 67 | 4 | **−** | + | + / + / ND | 2.700 | 1.053 | 1.323 | 0.621 | 16.5 | ↓ SN | PB | PB |
| 13. / F / 52 | 0 | + | − | + / − / + | 2.400 | 1.224 | 0.888 | 0.144 | 13.2 | N | PB | PB |
| 14. / F / 47 | 6 | + | + | + / + / + | 1.700 | 0.136 | 1.394 | 0.680 | 14.4 | ↓ BN and ↓ SN | PB, BM, Spleen | PB, Spleen |
| 15. / F / 61 | 7 | + | − | + / + / ND | 1.600 | 0.272 | 1.072 | 0.608 | ND | ND | PB | ND |
| 16. / F / 71 | 15 | ND | ND | N / + / + | 2.200 | 0.594 | 1.144 | 0.418 | ND | ND | PB | PB |
| 17. /M / 47 | 8 | − | + | N / + / + | 3.100 | 0.496 | 1.705 | 0.426 | 10.0 | ↓ SN | PB, BM | PB |
| 18. / F / 54 | 1 | − | + | + / + / + | 3.000 | 1.170 | 1.530 | 0.270 | 12.4 | N | PB, BM | PB |
| 19. / F / 30 | 5 | ND | + | + / + / ND | 1.100 | 0.430 | 0.420 | ND | 3.8 | ↓ SN | PB, BM | PB |
| 20. / F / 46 | 6 | + | − | + / + / ND | 3.400 | 1.100 | 1.800 | ND | ND | ND | PB | PB |
| 21. / F / 44 | 12 | + | + | + / + / ND | 1.900 | 0.399 | 1.254 | ND | ND | ND | PB | PB |
| 22. / F / 49 | 17 | + | + | + / + / + | 1.100 | 0.231 | 0.572 | ND | ND | ND | PB | PB |
| 23. / F / 69 | 8 | + | + | + / + / + | 1.500 | 0.390 | 1.020 | ND | 10.2 | ↓ SN | PB, BM | PB, BM |
| 24. / F / 59 | 7 | + | + | + / + / ND | 2.900 | 0.174 | 2.320 | ND | 12.2 | ↓ SN | PB, BM | PB |
| 25. / F / 57 | 0 | + | + | + / + / + | 1.200 | 0.400 | 0.600 | ND | ND | ND | PB, BM | PB, BM |

*, at the time of diagnosis of Felty's syndrome; y, years; RF, rheumatoid factor; anti-CCP, antibodies against cyclic citrullinated peptides; anti-MCV, antibodies against mutated citrullinated vimentin; ANA, antinuclear antibodies; BM, bone marrow; PB, peripheral blood; SN, segmented neutrophils; BN, band neutrophils; +, positive/present; −, negative/absent; ↓, reduction; N, normal; ND, no data; LGLs, large granular lymphocytes; STAT, signal transducer and activator of transcription gene
