# Supplementary material for: Analysis of a single-institution cohort of patients with Felty's syndrome and T-cell large granular lymphocytic leukemia in the setting of rheumatoid arthritis
Source: Rheumatol Int. 2020 Dec 5;41(1):147–56. doi: 10.1007/s00296-020-04757-4 (PMC7806571; doi:10.1007/s00296-020-04757-4)
Supplement: Supplementary file 3 — Supplementary file3 (DOC 132 KB) [file 296_2020_4757_MOESM3_ESM.doc]

**Supplement 3.** Characteristics of 56 patients with T-LGL leukemia and rheumatoid arthritis included in the study

| Patient no. / Sex / *Age (y) | *Duration  of RA (y) | Erosive arthritis | Splenomegaly | RF / anti-CCP / anti-MCV | Absolute  leukocyte count (×10^9^/L) | Absolute neutrophil count (×10^9^/L) | Absolute lymphocyte count (×10^9^/L) | Absolute LGL count (×10^9^/L) | Percent of lymphocytes in the BM | BM aspirate differential counting | Samples for T-cell clonality testing / T-cell clonality | Samples for STAT3 and STAT5b gene mutations testing / variants of STAT3 gene mutation |
| --- | --- | --- | --- | --- | --- | --- | --- | --- | --- | --- | --- | --- |
| 1**.** / F / 55 | 0 | − | + | + / + / + | 2.600 | 0.338 | 2.028 | 0.728 | 28.0 | ↓ BN , ↓ SN, ↓Met | PB / + | PB / p.Y640F |
| 2. / F / 60 | 11 | + | + | + / + / + | 2.900 | 0.812 | 1.653 | 0.551 | 9.0 | ↓ SN | PB / + | PB / p.Y640F |
| 3. / F / 43 | 4 | + | + | N / + / + | 1.600 | 0.016 | 1.197 | 0.544 | 22.8 | ↓ BN , ↓ SN, ↓Met | PB / + | PB / p.Y640F |
| 4. / F / 61 | 1 | − | + | N / + / ND | 2.700 | 1.188 | 1.269 | 0.621 | ND | ND | PB / + | PB / p.Y640F |
| 5**.** / F / 65 | 30 | + | − | + / + / ND | 6.290 | 1.394 | 2.898 | 1.890 | ND | ND | PB / + | PB / p.N647I |
| 6. / F / 65 | 12 | + | + | + / + / ND | 2.000 | 0.120 | 0.880 | 0.260 | 39.6 | ↓ BN , ↓ SN, ↓Met, ↓ Mye | PB / + | PB / − |
| 7. /F / 59 | 0 | + | + | N / + / ND | 1.300 | 0.208 | 0.780 | 0.338 | 21.2 | N | PB / + | PB / − |
| 8. / F / 43 | 5 | − | − | + / N / N | 3.000 | 1.160 | 1.250 | 0.387 | ND | ND | PB / + | PB / − |
| 9./ F / 70 | 0.5 | − | − | + / + / + | 4.700 | 0.752 | 3.572 | 1.739 | ND | ND | PB / + | PB / − |
| 10. / F / 66 | 35 | + | − | + / + / + | 2.400 | 0.312 | 1.728 | 0.864 | 39.5 | ↓ BN , ↓ SN, ↓Met, ↓ Mye | PB / + | PB / p.D661Y |
| 11. / M / 65 | 10 | + | − | + / + / ND | 0.790 | 0.836 | 2.332 | 0.954 | 34.8 | ↓ BN , ↓ SN, ↓Met | PB / + | PB / p.Y640F |
| 12. / F / 53 | 0 | − | − | + / + / ND | 3.700 | 1.332 | 1.776 | 0.592 | 41.6 | ↓ BN , ↓ SN, ↓Met, ↓ Mye | PB / + | PB / − |
| 13. / M / 56 | 0 | + | − | + / + / ND | 1.700 | 0.374 | 0.918 | 0.629 | ND | ND | PB / + | PB / − |
| 14. / F / 52 | 16 | + | − | N / + / + | 8.400 | 0.840 | 7.140 | 6.552 | 14.4 | N | PB / + | PB / p.N647I |
| 15. / F / 64 | 33 | + | − | + / + / N | 5.200 | 0.468 | 4.576 | 3.900 | 40.8 | ↓ BN , ↓ SN, ↓Met | PB / + | PB / p.D661Y |
| 16. / M / 63 | 1 | − | − | + / + / + | 1.700 | 0.408 | 1.258 | 0.510 | ND | ND | PB / + | PB / − |
| 17. / M / 67 | 6 | + | − | + / + / + | 5.500 | 0.825 | 4.235 | 2.805 | 80.3 | ↓ BN , ↓ SN, ↓Met, ↓ Mye | PB / + | PB / p.N647I |
| 18. / F / 62 | 1 | − | − | + / + / ND | 3.400 | 1.258 | 1.700 | 0.782 | ND | ND | PB / + | PB / − |
| 19. / F / 36 | 10 | + | + | + / + / ND | 2.100 | 0.126 | 1.827 | 1.029 | ND | ND | PB / + | PB / p.D661Y |
| 20. / M / 61 | 0 | + | + | + / + / ND | 3.000 | 0.900 | 1.900 | ND | 33.0 | ↓ SN | PB / +  BM / + | PB / − |
| 21. / F / 46 | 29 | + | + | + / + / ND | 4.400 | 0.868 | 3.432 | 2.332 | ND | ND | PB / + | PB / p.Y640F |
| 22. / M / 48 | 7 | + | + | + / + / + | 2.700 | 0.108 | 2.484 | 1.026 | 14.5 | ↓ SN | PB / +  BM / + | PB / − |
| 23**.** / F / 59 | 0 | + | − | + / + / + | 3.900 | 0.975 | 2.223 | 1.482 | 22.8 | ↓ BN , ↓ SN | PB / +  BM / + | PB / p.N647I |
| 24. / F / 48 | 21 | + | − | N / + / + | 7.900 | 1.185 | 6.557 | 6.162 | 16.6 | ↓ SN | PB / + | PB / − |
| 25. / F / 52 | 1 | + | + | + / + / + | 1.900 | 0.494 | 1.026 | 0.684 | 16.5 | ↓ SN | PB / +  BM / + | PB / −  BM / − |
| 26. / M / 49 | 16 | + | + | + / + / + | 1.600 | 1.078 | 0.814 | 0.374 | 8.1 | ↓ SN | PB / +  BM / + | PB / − |
| 27. /F / 47 | 12 | + | − | + / + / + | 4.500 | 0.495 | 2.880 | 1.215 | ND | ND | PB / + | PB / − |
| 28. / F / 72 | 6 | ND | + | + / + / + | 8.100 | 0.243 | 7.452 | 2.997 | ND | ND | PB / + | PB / − |
| 29. / F / 27 | 0 | − | + | + / + / + | 1.800 | 0.054 | 1.350 | 1.044 | 52.0 | ↓ BN , ↓ SN, ↓Met | PB / +  BM / + | PB / − |
| 30. / F / 48 | 3 | − | − | + / + / + | 7.200 | 3.096 | 3.888 | 2.376 | ND | ND | PB / + | PB / − |
| 31. / M / 62 | 0 | + | + | + / N / + | 2.500 | 0.100 | 2.075 | 0.850 | 71.6 | ↓ BN , ↓ SN, ↓Met | PB / +  BM / + | PB / − |
| 32. / F / 57 | 22 | + | + | + / + / + | 2.500 | 0.000 | 2.350 | 0.625 | 63.0 | ↓ BN , ↓ SN, ↓Met, ↓ Mye | PB / +  BM / + | PB / p.D661V  BM / p.D661V |
| 33. / F / 55 | 1 | − | − | + / N / N | 2.700 | 1.323 | 1.242 | 0.486 | 22.4 | N | PB / +  BM / + | PB / −  BM / − |
| 34. / F / 63 | 8 | ND | + | + / + / + | 0.700 | 0.056 | 0.630 | ND | 14.0 | ↓ SN | Spleen / +  BM / − | Spleen / − |
| 35. / F / 46 | 0 | − | + | + / + / + | 1.200 | 0.084 | 0.876 | 0.312 | 10.4 | ↓ SN | PB / +  Spleen / + | PB / −  Spleen / − |
| 36. / F / 48 | 6 | − | + | + / + / ND | 1.800 | 0.180 | 1.368 | ND | 13.2 | ↓ SN | PB / +  Spleen / + | PB / −  Spleen / − |
| 37. / M / 60 | 17 | + | + | + / + / ND | 1.400 | 0.154 | 0.756 | 0.560 | 10.0 | ↓ BN , ↓ SN | PB / −  Spleen / + | PB / −  Spleen / p.D661Y |
| 38. / F / 49 | 7 | − | + | + / + / + | 1.700 | 0.204 | 1.207 | ND | 62.6 | ↓ BN , ↓ SN, ↓Met | PB / +  Spleen / + | PB / p.Y640F  Spleen / p.Y640F |
| 39. / M / 39 | 0 | ND | + | + / + / + | 0.700 | 0.126 | 0.490 | 0.252 | 22.6 | ↓ BN , ↓ SN, ↓Met | PB / −  BM / −  Spleen / + | BM / −  Spleen / − |
| 40. / M / 62 | 0 | + | ND | N / N / N | 6.800 | 2.040 | 4.080 | 4.080 | 16.0 | ↓ BN , ↓ SN | PB / +  BM / + | PB / − |
| 41. / F / 65 | 13 | + | − | N / N / N | 3.700 | 1.332 | 1.924 | 0.814 | 17.6 | N | PB / +  BM / + | BM / − |
| 42. / F / 55 | 7 | + | − | N / + / N | 2.400 | 0.840 | 0.912 | 0.720 | ND | ND | PB / + | PB / − |
| 43. / F / 52 | 7 | ND | − | + / + / + | 5.600 | 1.400 | 2.816 | 1.920 | ND | ND | PB /+ | PB / p.D661Y |
| 44. / M / 54 | 1 | + | − | + / + / + | 7.700 | 1.463 | 5.852 | 5.467 | 35.2 | ↓ BN , ↓ SN, ↓Met | PB / + | PB / p.Y640F |
| 45. / F / 64 | 34 | + | − | + / + / + | 3.900 | 1.287 | 2.028 | 0.858 | 16.4 | N | PB / + | PB / p.N647I |
| 46. / M / 42 | 0 | − | + | + / + / + | 2.800 | 0.252 | 2.044 | 0.784 | ND | ND | PB / +  BM / + | PB / p.Y640F |
| 47. / M / 42 | 3 | − | + | + / + / + | 1.900 | 0.551 | 0.931 | ND | 7.8 | ↓ SN | PB / −  BM / +  Spleen / + | PB / −  Spleen / − |
| 48. / F / 68 | 8 | + | + | + / + / + | 1.970 | 0.177 | 1.281 | ND | 23.2 | ↓ SN | BM / +  Spleen / + | Spleen / − |
| 49. / M / 76 | 0 | + | + | + / + / ND | 2.700 | 0.066 | 1.936 | 0.660 | 16.2 | ↓ SN | PB / −  BM / +  Spleen / + | Spleen /p.N647I |
| 50. / F / 67 | 2 | − | + | + / + / + | 2.500 | 0.625 | 1.450 | ND | 8.4 | ↓ SN | PB / +  BM / + | PB / −  BM / − |
| 51. / F / 58 | 11 | + | − | N / N / N | 10.200 | 3.468 | 5.916 | 4.182 | 15.8 | N | PB / + | PB /− |
| 52. / F / 57 | 0 | + | − | N / N / N | 3.400 | 1.190 | 1.938 | 1.530 | ND | ND | PB / + | PB /− |
| 53. / F / 62 | 3 | − | + | + / + / + | 2.000 | 0.440 | 1.420 | 1.380 | 32.4 | ↓ BN , ↓ SN, ↓Met | PB / +  BM / + | PB / − |
| 54. / F/ 64 | 36 | + | + | + / + / + | 1.600 | 0.160 | 1.344 | 0.288 | 25.8 | ↓ BN , ↓ SN, ↓Met | PB / +  BM / + | PB / −  BM / − |
| 55. / F / 76 | 6 | + | + | + / + / + | 0.970 | 0.058 | 0.611 | ND | 21.6 | ↓ SN | PB / +  BM / +  Spleen / + | PB / p.D661Y  Spleen / p.D661Y |
| 56. / F / 69 | 16 | + | + | + / + / + | 1.100 | 0.176 | 0.550 | ND | 5.2 | ↓ SN | PB / −  Spleen / + | PB / −  Spleen / − |

*, at the time of diagnosis of T-LGL leukemia; y, years; RF, rheumatoid factor; anti-CCP, antibodies against cyclic citrullinated peptides; anti-MCV, antibodies against mutated citrullinated vimentin; ANA, antinuclear antibodies; BM, bone marrow; PB, peripheral blood; SN, segmented neutrophils; BN, band neutrophils; Met, neutrophilic metamyelocytes; Mye, neutrophilic myelocyte; +, positive/present; −, negative/absent; ↓, reduction; N, normal; ND, no data; LGLs, large granular lymphocytes; STAT, signal transducer and activator of transcription gene
